# Giant prolactinoma, germline *BRCA1* mutation, and depression: a case report

**DOI:** 10.1186/s13256-018-1890-x

**Published:** 2018-12-06

**Authors:** Rita Bettencourt-Silva, Joana Queirós, Josué Pereira, Davide Carvalho

**Affiliations:** 1 Department of Endocrinology, Diabetes and Metabolism, Centro Hospitalar Universitário de São João, E.P.E., Alameda Prof. Hernâni Monteiro, 4200-319 Porto, Portugal; 2 Faculty of Medicine, University of Porto, Alameda Prof. Hernâni Monteiro, 4200-319 Porto, Portugal; 3 Instituto de Investigação e Inovação em Saúde (i3S), University of Porto, Rua Alfredo Allen 208, 4200-135 Porto, Portugal; 4 Department of Neurosurgery, Centro Hospitalar Universitário de São João, E.P.E., Alameda Prof. Hernâni Monteiro, 4200-319 Porto, Portugal

**Keywords:** Prolactinoma, Prolactin, BRCA, Depression, Life events

## Abstract

**Background:**

Giant prolactinomas are very rare pituitary tumors that may exhibit an aggressive behavior and present with a life-threatening condition.

**Case presentation:**

A 25-year-old white woman was admitted to our hospital with a headache, psychomotor retardation, reduced vision, and loss of autonomy in daily activities. Her past medical history was significant for having oligomenorrhea and a depressive syndrome since her mother’s death. She also had a breast cancer gene 1 (*BRCA1*) mutation and a family history of breast cancer. She had marked hyperprolactinemia (7615 ng/dL), central hypocortisolism, growth hormone deficiency, and a giant pituitary tumor (52 × 30 × 33 mm) which was shown in magnetic resonance imaging with obstructive hydrocephalus, requiring emergency surgery. Treatment with cabergoline led to a 99.8% reduction in serum prolactin levels and significant tumor shrinkage. Her depressive symptoms progressively improved and psychiatric drugs were withdrawn after 3 months of cabergoline treatment. Currently, she is being followed in Endocrinology, Neurosurgery, and Neurophthalmology out-patient clinics and in a breast cancer unit. Careful monitoring, support, and follow-up will be essential throughout this patient’s life.

**Conclusions:**

This case is a rare presentation of a giant prolactinoma in a young woman, who presented a life-threatening event. She also had an unexpected association between diseases or symptoms that may have contributed to the delay in diagnosis. Given the concomitant presence of a giant prolactinoma, a *BRCA1* mutation, and depressive symptoms, a possible association was hypothesized. The breast cancer risk in a *BRCA1* mutation carrier and the possible interference of hyperprolactinemia and life events were also discussed. However this hypothesis requires further investigation.

## Background

Giant prolactinomas are pituitary adenomas larger than 4 cm in size presenting very high serum prolactin levels (typically above 1000 ng/mL) and no concomitant growth hormone (GH) or adrenocorticotropic hormone (ACTH) hypersecretion. They represent less than 5% of prolactin (PRL)-secreting tumors [1, 2]. Despite prolactinomas being more prevalent in women, giant prolactinomas are more common and are usually more aggressive in males (with an average male to female ratio of 9:1) [1], with only a few cases of giant prolactinoma being reported in females.

We describe a very rare giant prolactinoma in a young woman with breast cancer gene 1 (*BRCA1*) mutation and with depressive syndrome, who required an urgent surgical procedure for an unusual life-threatening acute hydrocephalus. The eventual relation between clinical presentation, PRL levels, depression, and *BRCA1* mutation will be discussed.

## Case presentation

### Present illness and previous history

A 25-year-old white woman presented to our Emergency Department (ER) in October 2014 with a 2-month history of headaches, morning vomiting, memory deficits, and sleepiness, followed by psychomotor retardation, reduced vision, and progressive loss of autonomy in daily activities during the preceding week. Her first menstruation occurred when she was 13-years old and her periods were regular until 15-years old, when she developed secondary amenorrhea and galactorrhea. She reported that in a previous evaluation she had high PRL levels and was submitted to pituitary imaging. These complementary studies were performed at another hospital and these results were not available. She was treated with bromocriptine for 2 years (from 15-years to 17-years old) with clinical improvement. Since then, she maintained oligomenorrhea without galactorrhea recurrence, but she missed the follow-up appointment. She also had grade 2 obesity with a body mass index (BMI) of 35.3 kg/m^2^ and was treated with fluoxetine and loflazepate ethyl for a severe depression since her mother’s death 4 years earlier. She was an asymptomatic *BRCA1* mutation carrier with heterozygous mutation c.2906delA (p.Asn969fs) in intensive cancer screening, because her family history was relevant for premature mortality due to breast cancer (mother and maternal aunt). At admission in our ER, she was hemodynamically stable, although a physical examination highlighted a depressive mood, marked psychomotor retardation, ataxia, and bilateral papilledema.

### Diagnostic assessment

**Fig. 1:**
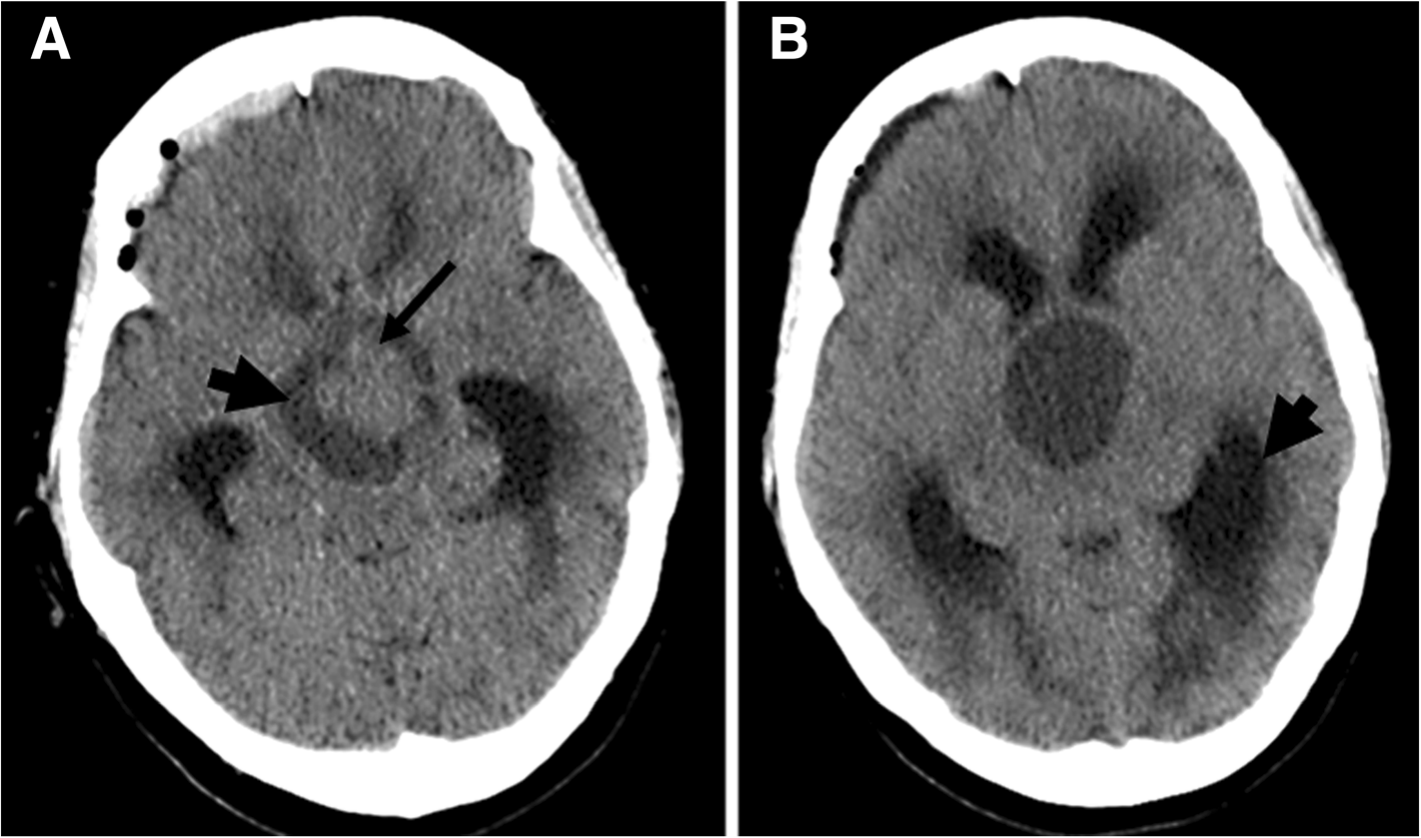
Computed tomography scan performed in Emergency Department. **a** Massive lesion with a solid component (*thin arrow*) and a cystic/necrotic component (*thick arrow*). **b** Obstructive hydrocephalus and dilatation of the lateral ventricles, especially on the left side (*thick arrow*)

Laboratory assessment at admission revealed normal values for the blood count (except mild leukocytosis), coagulation, liver and renal function tests, and electrolytes. There were no other signs of infection and urinary drug screening was negative. She underwent an urgent cerebral computed tomography (CT) scan, which revealed a giant pituitary lesion, with a cystic/necrotic component, extending to her sphenoid and cavernous sinuses (causing internal carotid artery compression) and suprasellar, prepontine, and interpeduncular cisterns. The lesion compressed her optic chiasma, basilar trunk, protuberance, and mesencephalon and obliterated the third ventricle, leading to obstructive hydrocephalus (Fig. 1). The results of a hormonal study were only available on the next day and revealed marked hyperprolactinemia (7615 ng/mL), central hypocortisolism, GH deficiency, normal thyroid function, and serum/urinary osmolality (Table 1).

**Table 1:** Endocrine assessment at baseline

Test	Result	Reference range
GH	**1.23** ng/mL	< 8 ng/mL
IGF-1	**131** ng/mL	219–644 ng/mL
TSH	0.72 μUI/mL	0.35–4.94 μUI/mL
FT4	1.09 ng/dL	0.70–1.48 ng/dL
FT3	3.33 pg/mL	1.71–3.71 pg/mL
PRL	**7615.0** ng/mL	4.8–23.3 ng/mL
Cortisol	**6.9** μg/dL	6.2–19.4 μg/dL
ACTH	**< 1.0** ng/L	< 63.3 ng/L
Serum osmolality	291 mOsmol/kg	282–300 mOsmol/kg
Urinary osmolality	313 mOsmol/kg	50–1200 mOsmol/kg

*ACTH* adrenocorticotropic hormone, *FT3* free triiodothyronine, *FT4* free thyroxine, *GH* growth hormone, *IGF-1* insulin-like growth factor 1, *PRL* prolactin, *TSH* thyroid-stimulating hormone

Significant alterations in endocrine assessment are indicated in bold

### Treatment

**Fig. 2:**
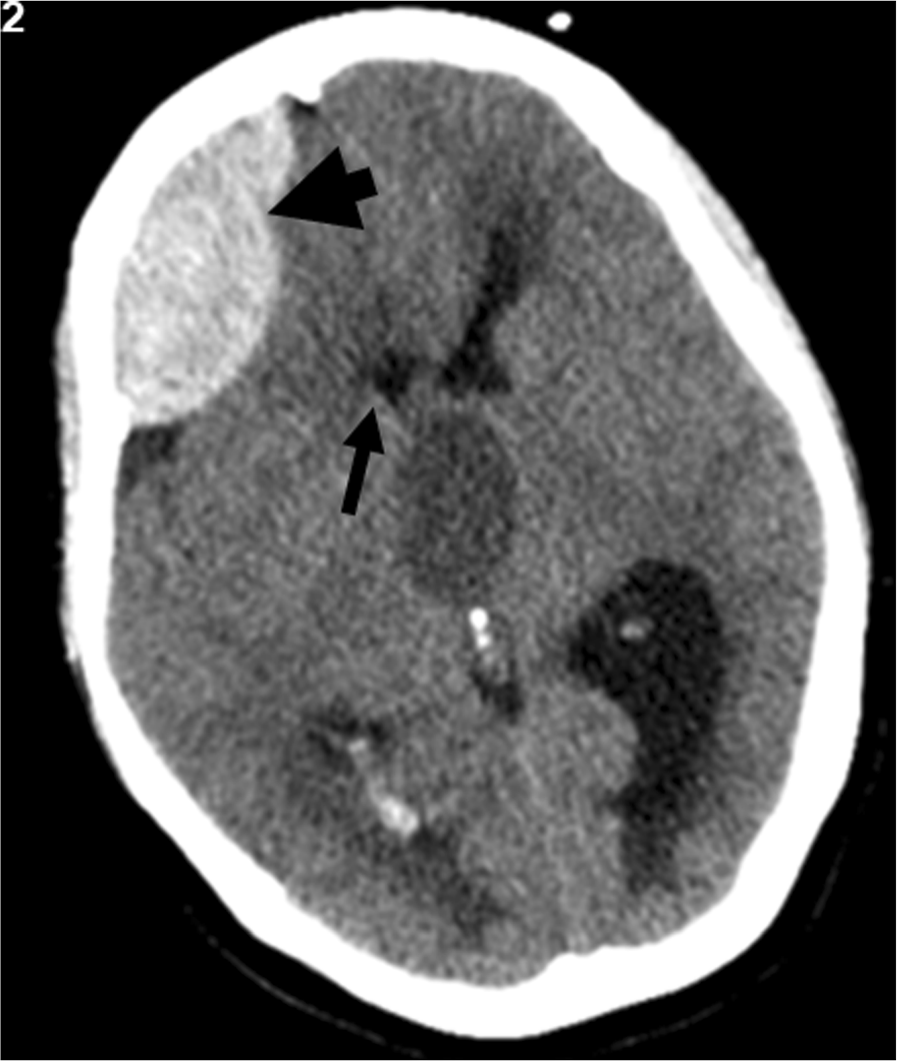
*De novo* postoperative extradural hematoma. Extradural hematoma was approximately 25.7 mm thicker (*thick arrow*) with a mass effect on the right frontal horn of the lateral ventricle (*thin arrow*)

Despite the probable pituitary etiology, endocrine assessment was in progress and our patient had a space-occupying brain lesion of still unknown origin and an acute hydrocephalus with rapid deterioration of consciousness. She was submitted to surgery for insertion of a right frontal external ventricular drain (EVD), but the early postoperative period was complicated by sudden-onset anisocoria and left upper limb paresis. A CT scan revealed a *de novo* large frontal extradural hematoma (Fig. 2), as a result of rapid decompression of the hydrocephalus. The hematoma was successfully removed by a new surgical procedure. Postoperatively, there was reversal of the new neurological deficits, without relapse or development of new symptoms.

**Fig. 3:**
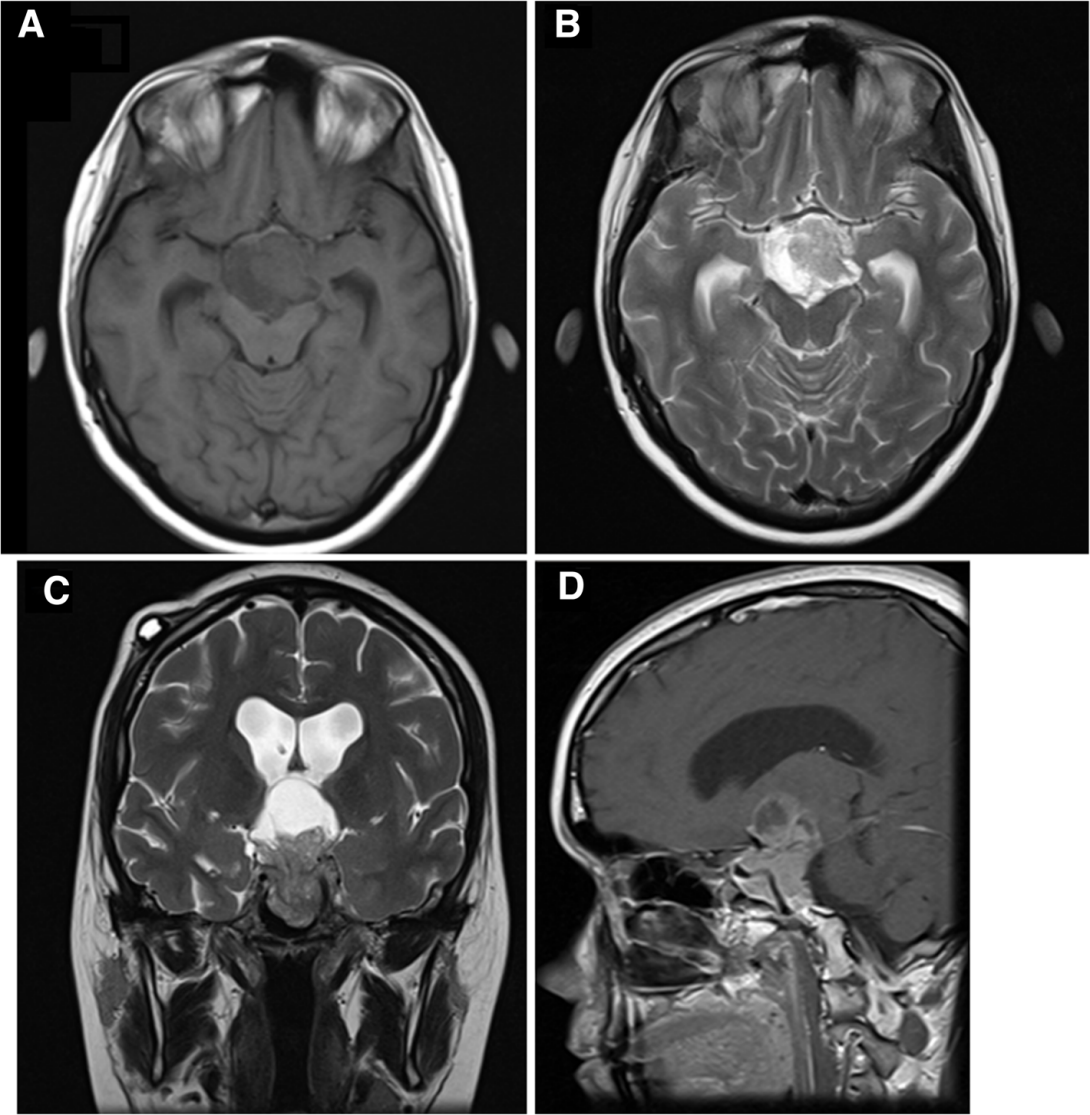
First pituitary magnetic resonance imaging with a giant prolactinoma. Axial T1 (**a**), axial T2 (**b**), coronal T2 (**c**) and sagittal T2-weighted fluid-attenuated inversion recovery images (**d**)

When she was observed by an endocrinology physician, she had already been started on intravenously administered dexamethasone 4 mg twice a day for cerebral edema. It was decided to keep this long-acting systemic corticosteroid and to switch to an orally administered hydrocortisone as soon as possible. Given the diagnosis of a large pituitary lesion with marked hyperprolactinemia, our patient was given orally administered cabergoline 0.5 mg twice a week. One week after admission, the EVD was substituted with a medium-pressure ventriculoperitoneal shunt, leading to an additional slight reduction in the dimensions of the lateral ventricles. After stabilization and 12 days of cabergoline, pituitary magnetic resonance imaging (MRI) confirmed a giant prolactinoma (52 × 30 × 33 mm in dimensions) with solid and cystic components, maintaining moderate residual supratentorial enlargement and confirming the massive invasion previously described in the CT scan (Fig. 3). During the in-patient management, short-term memory (assessed by Mini Mental State Examination) improved but our patient’s depressive mood was maintained. She was discharged on the 16th day under treatment with cabergoline 1 mg/week, hydrocortisone 30 mg/day in three divided doses (and progressive down-titration to 15 mg/day), fluoxetine 20 mg/day, and alprazolam 0.5 mg/day. She was referred to Endocrinology, Neurosurgery, and Neurophthalmology out-patient clinics.

### Follow-up and outcomes

One month after treatment with cabergoline, there was a 99.8% reduction in serum PRL levels (from 7615.0 ng/mL to 12.2 ng/mL). An ophthalmological examination performed only after discharge showed visual fields without signs of chiasmal compression. Two months after discharge, she underwent an ACTH stimulation test. Serum cortisol was 14.1 μg/dL and 43.9 μg/dL at baseline and after 60 minutes, respectively, excluding adrenal insufficiency; accordingly, hydrocortisone was withdrawn. Regarding the remaining pituitary hormonal profile, she had persistent GH deficit and central hypogonadism. Restoration of regular periods was achieved with an oral contraceptive pill. A mild elevated parathyroid hormone (PTH) level of 71.6 pg/mL (reference range 10–65) evoked the possibility of multiple endocrine neoplasia type 1 (MEN1). Bone densitometry revealed marked osteopenia of the lumbar spine with Z-score ≤ 2.0 (31% lower than expected). Total and ionized serum calcium levels were normal and PTH was normalized after vitamin D supplementation. However, genetic analysis was performed given the presence of an isolated sporadic giant prolactinoma in a young adult. *AIP* (encoding aryl-hydrocarbon receptor-interacting protein) and *MEN1* (encoding menin) sequencing (without multiplex ligation-dependent probe amplification) did not detect a pathogenic mutation.

Our patient’s depressive syndrome progressively improved and psychiatric drugs were withdrawn after 3 months of cabergoline treatment. She also lost 20 kg with lifestyle modification during the last 3 years (current BMI of 27.9 kg/m^2^), contributing to an improvement in overall wellbeing. Given the detection of a pathogenic *BRCA1* mutation, she started cancer screening at a young age in a breast cancer unit. Her screening combines breast self-examination, clinical breast examination and breast ultrasound, a mammography, and a transvaginal ultrasound. Up until now, the screening has been negative. Her sister is also a *BRCA1* mutation carrier under cancer screening. Currently, there is no familial history of hyperprolactinemia or pituitary lesions.

**Fig. 4:**
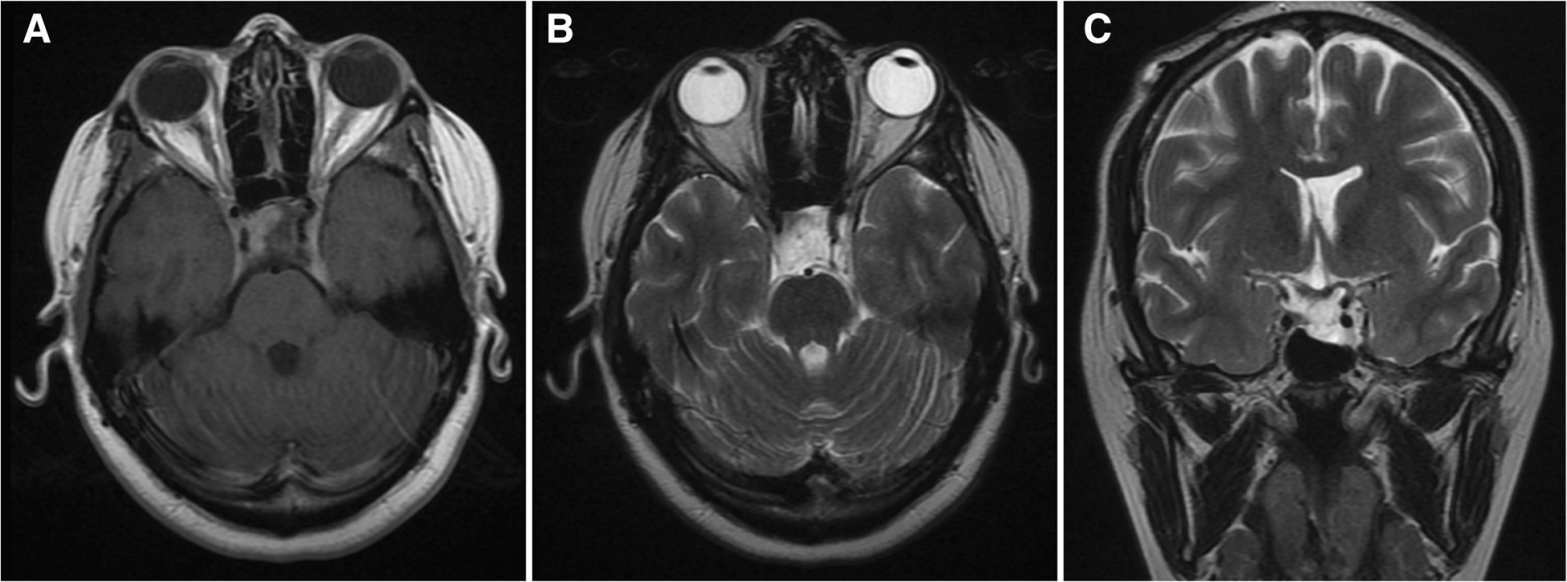
Last pituitary magnetic resonance imaging performed in September 2017. Pituitary tumor (20 × 15 × 20 mm) in axial T1-weighted fluid-attenuated inversion recovery (**a**), axial T2 (**b**) and coronal T2 images (**c**)

Treatment with cabergoline 0.5 mg twice a week was continued. The drug was well-tolerated and cardiac safety was assured by a transthoracic echocardiogram without evidence of valvular heart disease or any other heart dysfunction. The last pituitary MRI revealed a tumor measuring 20 × 15 × 20 mm (Fig. 4). She currently attends regular follow-up visits, with normal PRL levels and significant tumor shrinkage during this 3-year follow-up period.

## Discussion and conclusions

The clinical presentation of prolactinomas in women is commonly related to hyperprolactinemia (galactorrhea, oligomenorrhea/amenorrhea, decreased libido, and infertility), and giant prolactinomas can have a clinically aggressive behavior with mass effect (visual field defects, headaches, and rarely obstructive hydrocephalus). A recent review reported primary/secondary amenorrhea, visual impairment, and recurrent headaches as clinical features at diagnosis in 77%, 71%, and 59% of patients, respectively [1].

In addition, some patients with prolactinomas have depressive symptoms. PRL regulates a wide range of biological effects. Its secretion is stimulated by different stressors and influences human behavior, having an anti-depressive effect [3]. However, high levels of PRL can be converted to vasoinhibins in the hypothalamus after proteolytic cleavage, acquiring potent anxiogenic and depressive properties [3]. PRL exerts negative feedback on gonadotropin-releasing hormone in the hypothalamus, which controls pituitary pulsatile secretion of follicle-stimulating and luteinizing hormones. By this mechanism, or even by compression of the pituitary stalk, hyperprolactinemia results in decreased estrogen levels. Estrogen can play a role in serotonin activity and degradation, and low estrogen levels can contribute to the development of psychiatric disorders, such as depression [4]. Considering this association, low estrogen secondary to significant hyperprolactinemia from a giant prolactinoma may have contributed to our patient’s persistent and severe depressive mood. The concomitant presence of a depressive syndrome is probably one explanation for the acute presentation and delayed diagnosis of a space-occupying brain lesion, since memory impairment and sleepiness were given little value by some health care professionals. Furthermore, as she was depressed, our patient did not express her complaints.

Sonino *et al.* demonstrated a potential role of life events in the pathogenesis of both idiopathic hyperprolactinemia and prolactinomas [5]. The authors proposed that stress might facilitate clonal proliferation of a single mutated cell in prolactinomas. The onset of hyperprolactinemia-associated symptoms and signs can be temporally related to emotional stress, especially the loss of an important person. Interestingly, our patient lost her mother 4 years before the diagnosis of a giant prolactinoma was made. In addition, the confirmation of being a *BRCA1* mutation carrier was also an important cause for concern as our patient is a young woman. Whether the giant prolactinoma was related to our patient’s life events is unclear and seems difficult to prove, although a temporal association was observed.

Dopamine agonists should be the first-line treatment of giant prolactinomas [2]. Cabergoline has fewer side effects and is more effective than bromocriptine. The treatment led to visual field defects improvement, tumor volume reduction, and normalization of PRL levels in 96%, 74%, and 60% of patients, respectively [1]. In the case described above, the presence of previous psychiatric symptoms and a brain invasive space-occupying lesion without hormone assessment delayed the diagnosis and treatment. Cabergoline was safe, well-tolerated, and was very effective in this patient, with a 99% reduction in serum prolactin, significant tumor shrinkage, and an improvement of symptoms, hypocortisolism, and hypogonadism.

Molecular testing for genetic mutations has become an important tool. It has been suggested that all young patients (age ≤ 30 years) diagnosed with sporadic isolated pituitary macroadenomas, particularly prolactinomas, should be tested for both *MEN1* and *AIP* germline mutations [6].

The hypothesis that PRL can increase breast cancer risk has been discussed for many years. PRL can significantly contribute to breast oncogenesis, a role that was discussed in detail in a review by Clevenger *et al.* [7]. PRL receptors are highly over-expressed in malignant breast cancer tissue when compared with normal breast tissue. Several case reports of breast carcinomas in patients with prolactinomas have been reported [8–10]. Two case-control studies of the European Prospective Investigation into Cancer and Nutrition (EPIC) cohort were carried out regarding this issue. These studies found that higher circulating PRL levels were associated with an increased risk of *in situ* breast cancer among all women (both pre-menopausal and post-menopausal) [11] and an increased risk of breast cancer only in post-menopausal women (although the risk seemed to be confined to those who used postmenopausal hormone replacement therapy at blood donation) [12]. In a recent meta-analysis, Wang *et al.* supported a significantly positive association between plasma PRL levels and breast cancer risk among post-menopausal women, but not in pre-menopausal women [13]. This association was prominent for those with a positive estrogen and progesterone receptor cancer subtype. By contrast, some authors supported this association even in pre-menopausal women [14]. However, other studies did not find any association between breast cancer risk in women and hyperprolactinemia [15, 16]. In a long-term follow-up study, hyperprolactinemia was found to be an indicator of progressive disease and a poor prognosis in advanced metastatic breast cancer [17]. Furthermore, based on dopamine receptors over-expression in various breast tumoral cells, the use of cabergoline, which is a potent dopamine receptor agonist of D2 receptors, was found to induce apoptosis and necrosis in breast cancer cells and was recently proposed as a promising complementary therapy for breast cancer [18]. In addition, a phase II study of cabergoline in patients with metastatic breast cancer was carried out with a small subset of patients who had experienced extended disease control [19].

*BRCA1* and *BRCA2* genes are the most common genes associated with hereditary breast and ovarian cancer. The prevalence of pathogenic variants is estimated at 1:400–1:500 in the general population. Patients with a germline *BRCA1* mutation have a lifetime risk between 46 and 87% for breast cancer and between 39 and 63% for ovarian cancer [20]. Although no association was found between plasma PRL levels and *BRCA* carrier status among high-risk women, the presence of a giant prolactinoma associated with marked hyperprolactinemia in this patient could lead to an additional risk for the development of breast cancer. On the other hand, higher levels of PRL might also be associated with an increased risk of ovarian cancer [21].

Our hypothesis that prolactinomas may be associated with life events, depression, and increased risk of cancer in a *BRCA1* mutation carrier requires further research. Careful monitoring, support, and follow-up will be essential throughout this patient’s life.

### The patient’s perspective

“It all started with severe headaches and I just felt better lying down with everything dark. Then I started experiencing nausea and vomiting; there were days when I didn’t eat at all. My legs seemed to not want to walk and I stumbled many times and even fell. Slurred speech, difficult diction, memory loss and even urinary incontinence soon manifested themselves. I improved a lot after treatment, including my memory function. I have become a more calm and serene person and do not stress with things that used to upset me. I feel another person! Certainly I would not be alive if it wasn’t for the precious help of my father and sister, who took me to the hospital at the right time.”

## Abbreviations

ACTH: Adrenocorticotropic hormone
BMI: Body mass index
*BRCA1*: Breast cancer gene 1
CT: Computed tomography
ER: Emergency Department
EVD: External ventricular drain
GH: Growth hormone
MEN1: Multiple endocrine neoplasia type 1
MRI: Magnetic resonance imaging
PRL: Prolactin
PTH: Parathyroid hormone
